# Responses to balance challenges in persons with panic disorder: A pilot study of computerized static and dynamic balance measurements

**DOI:** 10.1002/brb3.2411

**Published:** 2021-11-29

**Authors:** Revital Amiaz, Shani Kimel Naor, Asaf Caspi, Efrat Czerniak, Shlomo Noy, Tatiana Pelc, Matti Mintz, Meir Plotnik

**Affiliations:** ^1^ Psychiatry Department The Chaim Sheba Medical Center Tel Hashomer Israel; ^2^ The Center of Advanced Technologies in Rehabilitation The Chaim Sheba Medical Center Tel Hashomer Israel; ^3^ Sackler Faculty of Medicine Tel Aviv University Tel Aviv Israel; ^4^ School of Psychological Sciences Tel Aviv University Tel Aviv Israel; ^5^ Sagol School of Neuroscience Tel Aviv University Tel Aviv Israel; ^6^ Department of Physiology and Pharmacology, Sackler School of Medicine Tel Aviv University Tel Aviv Israel

**Keywords:** dynamic balance, panic disorder, perturbations, static balance, sway

## Abstract

**Introduction:**

Several studies have shown an association between panic disorder (PD) and reduced balance abilities, mainly based on functional balance scales.

This pilot study aims to demonstrate the feasibility of studying balance abilities of persons with PD (PwPD) using computerized static and, for the first time, dynamic balance measurements in order to characterize balance control strategies employed by PwPD.

**Methods:**

Twelve PwPD and 11 healthy controls were recruited. PD diagnosis was confirmed using the Diagnostic and Statistical Manual of Mental Disorders, fourth edition (DSM‐IV), and the severity of symptoms was evaluated using the Hamilton Anxiety Scale (HAM‐A), PD Severity Scales (PDSS), and Panic and Agoraphobia Scale (PAS). Balance was clinically assessed using the Activities‐Specific Balance Confidence (ABC) scale and physically by the Mini‐Balance Evaluation Systems Test (Mini‐BESTest). Dizziness was evaluated using the Dizziness Handicap Inventory (DHI) scale. Postural control was evaluated statically by measuring body sway and dynamically by measuring body responses to rapid unexpected physical perturbations.

**Results:**

PwPD had higher scores on the HAM‐A (17.6 ± 10.3 vs. 3.0 ± 2.9; *p* < .001), PDSS (11.3 ± 5.1 vs. 0; *p* < .001), and PAS (20.3 ± 8.7 vs. 0; *p* < .001) questionnaires and lower scores on the balance scales compared to the controls (ABC scale: 156.2 ± 5.9 vs. 160 ± 0.0, *p* = .016; Mini‐BESTest: 29.4 ± 2.1 vs. 31.4 ± 0.9, *p* = .014; DHI: 5.3 ± 4.4 vs. 0.09 ± 0.3, *p* < .001). In the static balance tests, PwPD showed a not‐significantly smaller ellipse area of center of pressure trajectory (*p* = .36) and higher body sway velocity (*p* = .46), whereas in the dynamic balance tests, PwPD had shorter recovery time from physical perturbations in comparison to controls (2.1 ± 1.2s vs. 1.6 ± 0.9 s, *p* = .018).

**Conclusion:**

The computerized balance tests results point to an adoption of a ‘‘postural rigidity’’ strategy by the PwPD, that is, reduced dynamic adaptations in the face of postural challenges. This may reflect a nonsecure compensatory behavior. Further research is needed to delineate this strategy.

## Graphical abstract

In our study we challenge the association between panic disorder (PD) and reduced balance abilities demonstrated in previous studies. In this study we used computerized static and, for the first time, dynamic balance measurements. Our balance assessments results show that PD patients subjectively feel unstable while they are mechanically more stable than controls.

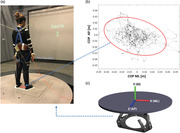

## 1 INTRODUCTION

Panic disorder (PD) is a common psychiatric disorder with a lifetime prevalence of 4.7% (Goodwin et al., 2005), characterized by recurrent unexpected panic attacks (for review see Kessler et al., 2006).

### 1.1 Comorbidities between PD and vestibular and balance control impairments

Many studies have shown an association between PD and vestibular symptoms (e.g., dizziness, vertigo, or imbalance). Up to 10% of persons referred to otolaryngology clinics suffer from psychogenic disorders, including PD, who exhibit twofold greater prevalence than the general population (Asmundson et al., 1998; Clark et al., 1994; Feldman et al., 2019; Jacob et al., 1996; Perna et al., 2001; Simon et al., 1998; Staab, 2006; Staab & Ruckenstein, 2005; Stambolieva & Angov, 2010; Yardley et al., 1995, 2001).

Some studies observed that persons with PD (PwPD) suffer from objective otolaryngologic symptoms (Staab, 2013; Staab & Ruckenstein, 2005; Teggi et al., 2007), and others reported that PwPD do not have specific vestibular impairments but have minor signs of balance abnormalities and impairments in balance reactivity to visual stimuli (Asmundson et al., 1998; Caldirola et al., 2011; Jacob et al., 1997; Perna et al., 2001; Staab, 2006).

### 1.2 Objective assessment of balance control

Balance control is a complex process that requires sensory‐motor integration, for example, between vestibular, visual, proprioception, and motor control systems (Angelaki & Cullen, 2008). Frequently used clinical balance rating scales are susceptible to tester bias or to patients’ subjective self‐report (Mancini & Horak, 2010; Stambolieva & Angov, 2010; Wood et al., 2015). Thus, in recent years, there has been a notable advancement in several technologies used for objective and precise measurement of balance control, including the one at the focus of the present study, that is, posturography and moving surfaces (Brauer et al., 2002; Mok & Hodges, 2013; Redfern et al., 2002).

Static balance is evaluated by measuring the postural sway (i.e., the slight body sway naturally occurring while standing), which is usually quantified by characterizing displacements of the center of pressure (CoP), extracted from force plate sensors on the standing surface (Lajoie & Gallagher, 2004; Perna et al., 2001). CoP ellipse area and CoP velocity were both found to be indicative of balance control (Kim et al., 2009; Tanaka et al., 2002).

Dynamic balance measurements require first to unexpectedly destabilize the participant by physical perturbations applied on the standing surface, that is, horizontal or vertical translations, rotations, or tilts (Abboud et al., 2016; Porras et al., 2021; Wood et al., 2015). This kind of methodology provides distinctive information about the participant's balance‐recovery abilities.

### 1.3 Objective assessment of balance control in PwPD and in healthy participants—The "rigidity" strategy during anxiety and threatening conditions

Posturographic evaluation of PwPD has focused mainly on static balance indices. For example, it has been observed that PwPD have small sway area and higher CoP velocity when standing on hard surfaces (Lopes et al., 2009). When adding a nonthreatening interference, PwPD have larger sway area and higher CoP velocity (Caldirola et al., 2011; Redfern et al., 2007; Stambolieva & Angov, 2010, 2009), Based on the findings of Lopes et al. (2009), PwPD use a postural “rigidity” strategy for maintaining balance, that is, preference to reduce mobility.

In a related research, healthy participants were exposed to anxiety‐causing situation by instructing persons to stand on elevated surfaces, while posturography was measured. Postural “rigidity” or “stiffening” strategy was reported (Carpenter et al., 1999), by expressing smaller CoP areas during static standing with the increase in the anxiety level (Adkin & Carpenter, 2018), where the behavior can be interpreted as cautious‐related rigidity. An additional related recent body of research specifically addressed postural (and emotional) behavior in healthy participants in relation to anxiety induced by the threat of an imminent physical surface perturbation. It was observed that when anticipating dynamic surface perturbations, a situation that is perceived as a “threat,” static sway was actually increased (Bax et al., 2020; Johnson et al., 2019a, 2019b; Phanthanourak et al., 2016). It was argued that although rigidity may be beneficial during static tasks, such postural control strategy may be detrimental during dynamic balance tasks (e.g., in the case of fear of falling; for review, Young & Williams, 2015).

During dynamic perturbations, high anxiety levels were expressed by faster reaction of the body as reflected by the CoP, center of mass (CoM) (Brown & Frank, 1997; Carpenter et al., 2004; Cleworth & Carpenter, 2016; Okada et al., 2001), or muscles activity (Adkin et al., 2008; Cleworth & Carpenter, 2016; Okada et al., 2001). The CoP displacement increased during the exposure to a perturbation in high anxiety conditions (Cleworth & Carpenter, 2016; Okada et al., 2001), while the CoM displacement decreased (Brown & Frank, 1997; Carpenter et al., 2004).

### 1.4 Study rational and objectives

In light of the relatively sparse literature describing objective postural control measures in dynamic conditions in PwPD, and in light of the aforementioned findings that postural rigidity is observed in PwPD during static tasks but not by healthy participants anticipating the “threat” of physical perturbation, we propose to further study the anxiety–postural control relation by focusing on dynamic postural control response (i.e., to physical perturbations) in PwPD.

It has been well established that deprived vision impairs balance control e.g., as expressed by increased static sway (Cavalheiro et al., 2009; Hideyuki et al., 2002; Woollacott & Shumway‐Cook, 2002). In addition, it has been shown that performing a secondary cognitive task while maintaining balance might have a detrimental effect (e.g., Brauer et al., 2001; Brown et al., 1999; Woollacott & Shumway‐Cook, 2002). These effects have been hardly studied in the context of PwPD (but see, Perna et al., 2001; Redfern et al., 2007; Stambolieva & Angov, 2010).

The objective of the present study was to conduct a pilot trial to test, for the first time, the feasibility of evaluating if potential postural "rigidity" strategy employed by PwPD (Lopes et al., 2009) is expressed during dynamic balance challenges in PwPD comparing to healthy controls. Feasibility assurance was needed in order to ascertain whether PwPD can tolerate repeated presentation of physical perturbations.

Further, in order to address the conflicting findings from static balance control in these cohorts (see above), we also evaluated static sway. In particular, we tested the following predictions: (1) during static sway, the CoP displacement of PwPD will be smaller compared to controls and (2) in response to physical perturbations, PwPD will have increased CoP displacement and velocity as compared to healthy controls.

## 2 METHODS

### 2.1 Participants

We attempted to recruit 12 PwPD and 12 healthy controls for this pilot trial. Twelve PwPD (age: 36.9 ± 10.3 years, four men) and 11 healthy controls (age: 31.8 ± 4.3 years, six men) with similar physical and demographic characteristics (see Table 1) participated in the study. Sample size calculation was estimated using WinPepi (version 11.65). Comparison of means was used with CoP displacement during perturbations in young healthy adults standing on low‐ and high‐level platforms (increased anxiety condition) obtained from the study of Cleworth et al. (2016), and an expected difference in CoP displacement of 2.8 [mm] was postulated (Cleworth & Carpenter, 2016). Power analysis showed that for effective detection in a two‐group design with 80% power and *α* = .05, a total sample size of 12 is required (six in each group). Based on this result, we decided to take a conservative approach and target sample size twice as big (i.e., 12 participants in each group). We also considered a dropout rate of ∼15%. However, this power calculation does not address the multiple conditions in this pilot trial (see below and in Section 4.4).

**TABLE 1:** Demographic and clinical data of panic disorder (PD) versus healthy participants

	Type	Healthy (mean ± SD)	PD (mean ± SD)	*p*‐Value*
Demographic	No. of participants	11	11**	N.S. (1)
	Age (y)	31.3 ± 6.3	36.9 ± 10.3	N.S. (.20)
	BMI (kg/m^2^)	22.3 ± 3.3	25.4 ± 6	N.S. (.29)
Anxiety level	HAM‐A score (x/52)	3.0 ± 2.9	17.6 ± 10.3	<.001
	PDSS score (x/28)	0	11.3 ± 5.1	<.001
	PAS	0	20.3 ± 8.7	<.001
	CGI score (x/7)	1.0 ± 0	3.4 ± 0.9	<.001
	VAS score (x/10)	0.6 ± 1.1	1.3 ± 1.9	N.S. (.38)
Functional balance	ABC score (x/160)	160.0 ± 0.0	156.2 ± 5.9	<.016
	DHI score (x/25)	0.1 ± 0.3	5.3 ± 4.4	<.001
	Mini‐BESTest score (x/32)	31.4 ± 0.9	29.4 ± 2.1	<.014

Abbreviations: BMI, body mass index; ABC, Activities‐Specific Balance Confidence; CGI, Clinical Global Impression scale; DHI, Dizziness Handicap Inventory; HAM, Hamilton Anxiety Scale; Mini‐BESTest, Mini‐Balance Evaluation Systems Test; PAS, Panic and Agoraphobia Scale; PDSS, PD Severity Scale; VAS, Visual Analog Scale.

* Nonparametric Mann–Whitney *U*‐test between panic disorder and healthy participants. ** One participant disqualified (see text).

Participants were recruited from the Psychiatric Clinic of the Rehabilitation hospital of Sheba Medical Center, Ramat Gan, Israel. Patients were stabilized for at least 1 month prior to the study (seven PwPD took antidepressants, one patient took oxazepam in addition). Seven patients were suffering from agoraphobia in addition to PD. Patients who also suffered from cognitive dysfunction, neurological disorders, vestibular disorders, or major psychiatric disorders in addition to PD were excluded. One patient was disqualified from the study due to a panic attack during the assessment. The protocol was approved by the institutional review board at Sheba Medical Center.

### 2.2 Apparatus

**FIGURE 1:**
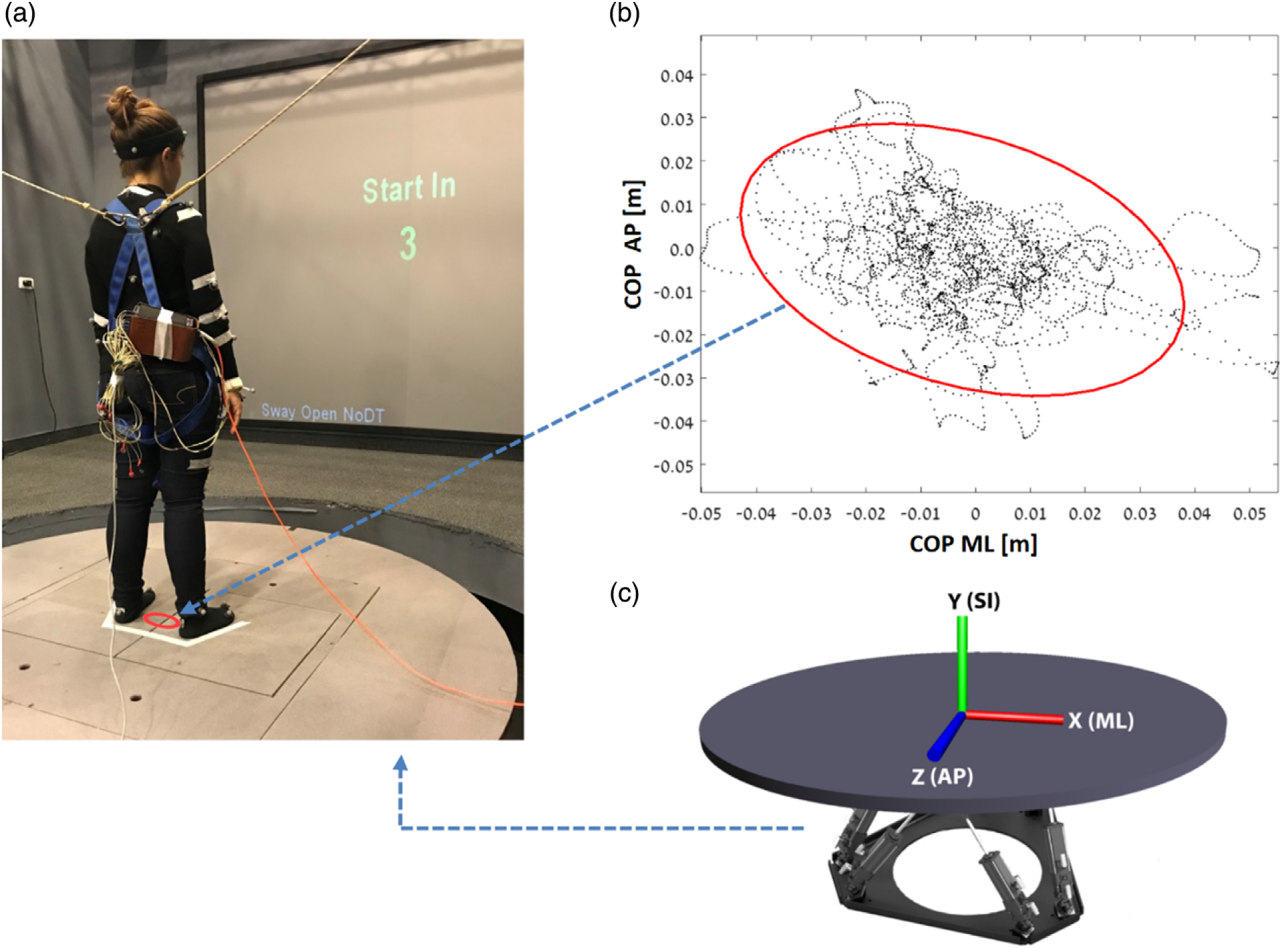
(a) Participant sway on a platform with two embedded force plates tracking her center of pressure displacement (Dc_o_p); (b) Dc_o_p area in the medial‐lateral (ML) and anterior‐posterior (AP); (c) A moveable platform with six degrees of freedom illustration (*x*, *y*, and *z* axes). The platform is synchronized with a motion capture system (Vicon, Oxford, UK), which collects kinematic positional data from 41 passive reflective markers attached to the participant's body (120 Hz; 1 mm resolution). Electrocardiography (ECG) was recorded as well. Kinematic and ECG results are not presented here. The weighted Dc_o_p was calculated by using the distance equation $D_{\mathrm{CoP}}=\sqrt{{D_{\mathrm{CoP}}}_{\mathrm{AP}}^{2}+{D_{\mathrm{CoP}}}_{\mathrm{ML}}^{2}}\;$

A movable circular platform (radius = 2 m), which is a part of large‐scale virtual reality system (CAREN‐Base, Motek Medical, The Netherlands), was used. The platform can move with six degrees of freedom, with two force plates embedded in it (120 Hz; 1 mm resolution). Synchronized video cameras recoreded the experiments. A plus sign was projected on the screen as a visual fixation point, located 2.3 m from the participant (see Figure 1).

### 2.3 Procedure

After providing written informed consent, the participants were evaluated with four types of assessments in the following order: psychiatric questionnaires, clinical functional balance rating scales, and computerized static and dynamic balance measurements.

#### 2.3.1 Psychiatric questionnaires

Participants’ diagnosis was assessed using the Mini International Neuropsychiatric Interview (MINI) and was validated using the Structured Clinical Interview for the Diagnostic and Statistical Manual of Mental Disorders, fourth edition (DSM‐IV; SCID‐1). Severity of panic symptoms was evaluated using the Hamilton Anxiety Scale (HAM‐A), PD Severity Scale (PDSS), Panic and Agoraphobia Scale (PAS), and the Clinical Global Impression (CGI) scale. Severity of anxiety at the time of evaluation was measured using a visual analog scale (VAS).

#### 2.3.2 Clinical functional balance rating scales

The participants’ subjective balance assessment was obtained using the Activities‐Specific Balance Confidence (ABC) scale and Dizziness Handicap Inventory (DHI). In addition, functional balance was physically evaluated by the Mini‐Balance Evaluation Systems Test (Mini‐BESTest). To simulate more every day like situation, we added a test condition during which cognitive load was applied as a dual task (DT) of serial 7‐subtraction from randomized three‐digit numbers (e.g., 500, 493, 486, etc.). This cognitive task was chosen to simulate physical effort during constant cognitive concentration (e.g., walking while talking on the cellphone). Dual tasking was performed during both static and dynamic measurements. Prior to starting the balance control trials (static and dynamic), we recorded a baseline period per participant in which they performed the counting task solely, which then served as the reference.

#### 2.3.3 Static balance measurement

Participants were instructed to stand comfortably on a platform in a convenient posture, legs spread to the width of their shoulders and feet in outward rotation of approximately 15° from the midline (Hof et al., 2005). The initial feet placement of each participant was marked on the surface using removable tape. Participants were instructed to return and resume this placement if they have shifted during the experiments. The computerized static balance measurements included 30 s of quiet standing (i.e., “sway”) on the platform for four conditions, in a random order: eyes open (EO), eyes closed (EC), and each during the presence/absence of the DT (Yardley et al., 1999).

#### 2.3.4 Dynamic balance measurement

The computerized dynamic balance measurements included two standing trials during which physical perturbations were introduced, with and without DT (NoDT). In each trial, the participant was asked to stand on the platform while keeping their balance and eight sudden perturbations were applied to the platform in succession and in a random order: translated and tilted forward, backward, left, and right. Five seconds after each perturbation, the platform slowly returned to its initial position, followed by a period of time randomly varied from 9 to 15 s, until the next perturbation occurred. Translation perturbations were performed by a 10 cm platform movement over 0.3 s, and tilt perturbations were performed by a 5° platform tilt over the earth vertical axis over 0.3 s. Participants returned to their initial marked position on the platform in the case of a stepping response.

We also assessed static balance during the periods (5 s duration) of standing prior to the introduction of each dynamic perturbation (Johnson et al., 2019b; Stins & Beek, 2011). This was done in order to assess the potential effect of task performance in repose to the perturbation.

### 2.4 Analysis and outcome measures

**FIGURE 2:**
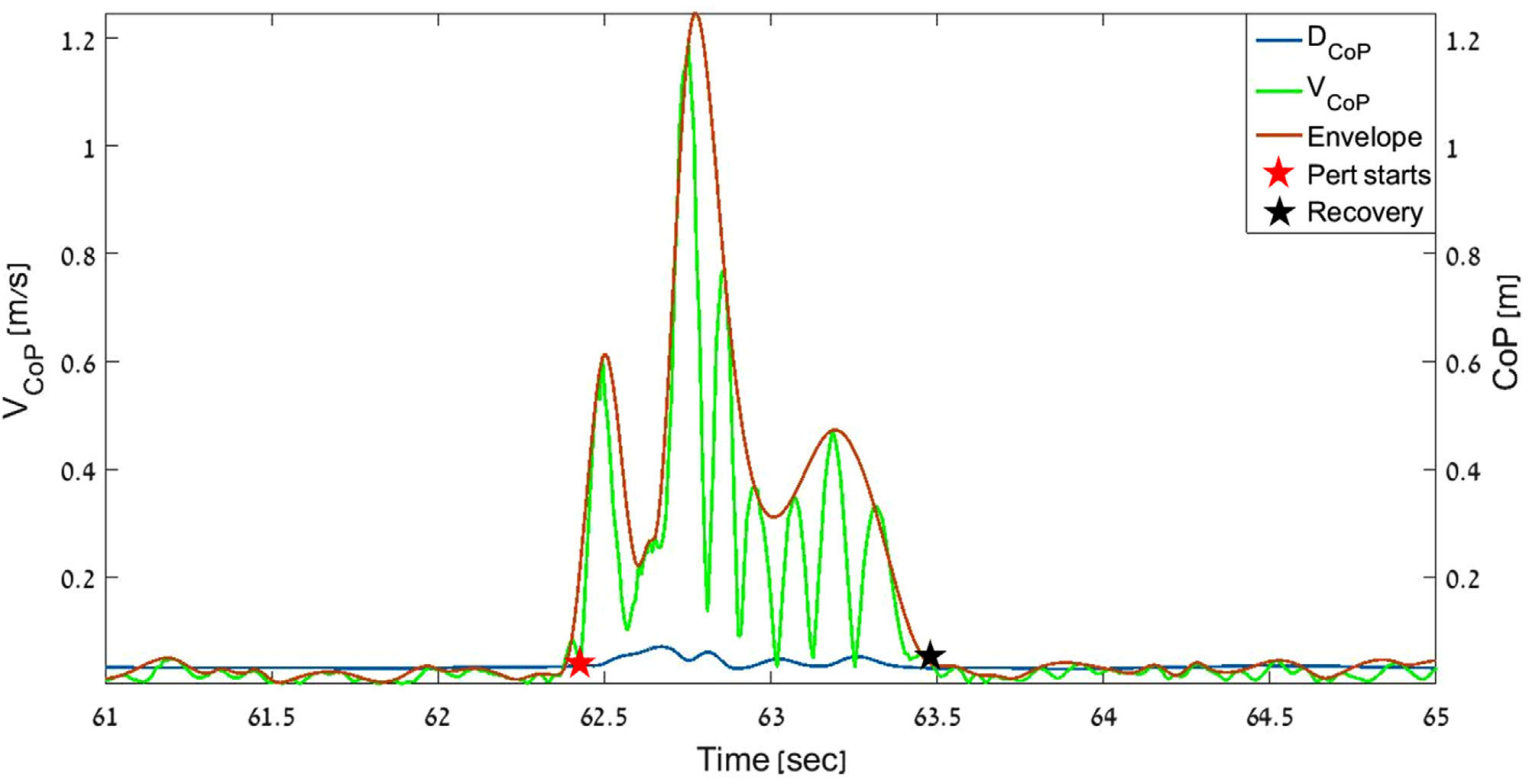
Center of pressure displacement (Dc_o_p; blue) and absolute velocity ($\bar{V_{\mathrm{CoP}}}$; green) during a left rotation perturbation with dual task (DT) time segment, from a single session of a single participant. An “envelope” over the velocity signal is shown in red. A red star marks onset of the perturbation (Pert), and a black star marks the participant's recovery

The system extracts the CoP coordinates from the two force plates, thus enabling calculation of a weighted CoP displacement (Dc_o_p) from both legs in the anterior‐posterior (AP) and the medial‐lateral (ML) axes. The weighted CoP was calculated by using the distance equation (Figure 1). Center of pressure velocity ($\bar{V_{\mathrm{CoP}}}$), was defined as the Dc_o_p derivative over time (Figure 2). All parameters were analyzed using custom developed algorithms applied in MATLAB (MathWorks, Inc.).

#### 2.4.1 Static balance analysis

Participants’ postural stability, which was taken from the static balance measurement (during sway periods and standing periods prior to the introduction of perturbations), was evaluated using the Dc_o_p, and incuded: (1) Dc_o_p area, measured as an ellipse area covering 95% of the two‐dimensional Dc_o_p (Figure 1b), (2) average velocity ($\bar{V_{\mathrm{CoP}}}$), measured during the middle 20 s of each condition. Balance stiffness is usually reflected by a small Dc_o_p ellipse area and high $\bar{V_{\mathrm{CoP}}}$ during different sway conditions.

#### 2.4.2 Dynamic balance analysis

Participants’ balance abilities, which were taken from the dynamic balance measurement trials, were analyzed over separate time segments, starting 5 s before and ending 4.5 s after each perturbation. The participants’ postural responses to the perturbations were evaluated by analysis of the Dc_o_p, and included: (1) recovery time, calculated as the time to return to a steady state after the perturbation, (2) Dc_o_p maximal value, and (3) averaged velocity during the recovery time (Brauer et al., 2001). Low balance abilities were found to be correlated with slower recovery time after perturbations, stiffer body responses, and the use of multiple steps for recovery. Representative output of a single perturbation in an individual participant is shown in Figure 2.

#### 2.4.3 Response strategy analysis

We differentiated between two strategies of balance‐recovery from perturbations: in‐place and step responses. This differentiation was performed based on the pressure magnitude recorded by the two force plates and on visual cues from the videos. A recovery step was defined as zero pressure from one foot, that is, the foot is in the air for at least 50 ms while causing body movements that affected the size of the body's base of support (Brown et al., 1999). The step's total displacement was calculated similarly to the Dc_o_p calculation, and the number of steps needed to recover balance were counted.

#### 2.4.4 Dual task cost

The cost of cognitive attention task on stability was analyzed in both static and dynamic balance conditions. To quantify this, we calculated the dual task cost (DTC) on balance performance, which measures the percent change of balance performance on dual task from single task performance (Bock, 2008).

(1) $$\begin{array}{ccc} \mathrm{DTC} & = & 100\times (\mathrm{single}-\mathrm{task}\;\text{score-dual-task}\;\text{score})/\\ & & \mathrm{single}-\;\text{task}\;\text{score} \end{array}$$

We also calculated cognitive dual task costs (C‐DTC) as follows: we computed the counting rate (CR) during the baseline (“reference”) counting (see Section 2.3.2), during the 30 s long static sway measurements, and during the dynamic perturbations (this period could vary within participants, see Section 2.3.4). From these data, we could extract the following C‐DTC:

(2) $$\begin{array}{c} \text{Sway}\;\text{C-DCT}=100\times \left(\text{reference}\;\text{CR}-\mathrm{Sway}\;\mathrm{CR}\right)/\mathrm{reference}\;\mathrm{CR} \end{array}$$

(3) $$\begin{array}{ccc} \text{Perturbation}\;\text{C-DCT} & = & 100\times (\text{reference}\;\text{CR}-\;\text{Perturbation}\;\text{CR})/\\ & & \text{reference}\;\text{CR} \end{array}$$

(4) $$\begin{array}{ccc} \text{Sway}\;\text{vs.}\;\text{Perturbation}\;\text{DCT} & = & 100\times (\text{sway}\;\text{CR}-\;\text{Perturbation}\;\text{CR})/\\ & & \text{sway}\;\text{CR} \end{array}$$

### 2.5 Effect of agoraphobia

In previous studies, agoraphobia has been highly correlated with balance disturbances (Jacob et al., 1996; Perna et al., 2001; Yardley et al., 1995, 2001). Based on our clinical records, we identified an agoraphobia and nonagoraphobia subgroups within the PwPD group, and compared their balance performances.

### 2.6 Statistical analysis

Due to relatively small sample sizes, we used a nonparametric Mann–Whitney test to compare balance abilities between PwPD and healthy controls across all assessments. Outliers were removed when values were beyond 1.5 interquartile range from the median. Statistical significance was defined at *p* < .05.

## 3 RESULTS

### 3.1 Clinical assessments

Table 1 depicts the participants’ demographic data, scores of the psychiatric questionnaires, and functional balance rating scales. Participants with PD had significantly higher scores on the anxiety level evaluations (i.e., more anxiety) and significantly lower scores on the functional balance assessments (i.e., less confidence in their balance, dizzier, and less stable). The VAS score was recorded prior to the physical protocol, and the results showed that all participants, except one, started the experiment at a low anxiety level.

### 3.2 Static balance

In the static balance assessment, significant differences were found between the groups in a few CoP parameters while others pointed on trends, although not statistically significant, as presented in Table 2. The Dc_o_p ellipse area tended to be smaller in the PD group for sway in EO (N.S.) and EC (*p* = .004) conditions. The $\bar{V_{\mathrm{CoP}}}$ tended to be higher in the PD group for sway in EO and EC conditions, but with no statistically significant group effect.

**TABLE 2:** Computerized static and dynamic measurement results of panic disorder (PD) versus healthy participants

		Parameter	Healthy (mean ± SD)	PD (mean ± SD)	*p*‐Value*
Sway	Ellipse area of Dc_o_p	EO (mm^2^)	148.2 ± 116.1	105.8 ± 90.5	N.S. (.36)
		EC (mm^2^)	161.4 ± 39.6ª	87.0 ± 50.7ª	.004
		EO DT (mm^2^)	630.4 ± 531.3ª	405.5 ± 386.7	N.S. (.28)
		EC DT (mm^2^)	279.4 ± 172.8ª	183.0 ± 146.1ª	N.S. (.12)
		DTC EO (%)	−858.1 ± 1073.7ª	−214.8 ± 224.7ª	N.S. (.24)
		DTC EC (%)	−66.6 ± 119.9^b^	−77.4 ± 109.7^b^	N.S. (.60)
	$\bar{V_{\mathrm{CoP}}}$	EO (mm/s)	9.1 ± 2.2ª	12.8 ± 8.3	N.S. (.46)
		EC (mm/s)	10.8 ± 1.1^b^	11.7 ± 4.1ª	N.S. (.90)
		EO DT (mm/s)	22.8 ± 9.7	15.5 ± 6.4^b^	N.S. (.07)
		EC DT (mm/s)	18.3 ± 6.6	13.1 ± 2.6^b^	.040
		DTC EO (%)	−157.3 ± 91.9^a^	−50.8 ± 39.6^b^	.017
		DTC EC (%)	−62.1 ± 42.6	−27.5 ± 26.0	N.S. (.39)
Perturbations^†^	In‐place	Recovery time (s)	1.24 ± 0.6	1.23 ± 0.6	N.S. (.95)
		CoP max (m)	0.13 ± 0.04	0.14 ± 0.05	N.S. (.94)
		$\bar{V_{\mathrm{CoP}}}$ (m/s)	0.087 ± 0.04	0.10 ± 0.05	.015
		$\bar{V_{\mathrm{CoP}}}$ (mm/s)	87.0 ± 43.1	99.2 ± 50.4	.015
	Steps	Step recovery type (%)	38.6 ± 22.0	36.4 ± 20.5	N.S.
		Multiple steps episodes (#)	1/7	6/7	.02 (*χ* ^2^)
		Recovery time (s)	2.1 ± 1.2	1.6 ± 0.9	.018
		Total displacement (cm)	9.3 ± 7.9	12.9 ± 12.0	N.S. (.17)
	Standing prior	Ellipse area of Dc_o_p (mm^2^)	654.6 ± 528.9	699.3 ± 664.8	N.S. (.87)
		$\bar{V_{\mathrm{CoP}}}$ (mm/s)	30.7 ± 16.5	38.5 ± 23.9	N.S. (.43)

*Note*: Dc_o_p is the center of pressure displacement, $\bar{V_{\mathrm{CoP}}}$ is the averaged center of pressure velocity.

Abbreviations: DT, dual task; DTC, dual‐task cost; CoP, center of pressure; EC, eyes closed; EO, eyes open.

^a^ One outlier removed.

^b^ Two outliers removed.

* Nonparametric Mann–Whitney *U*‐test between panic disorder (PD) and healthy participants.

^†^ All types of perturbations were statistically tested separately; mean values presented are the values for all perturbations types together.

**FIGURE 3:**
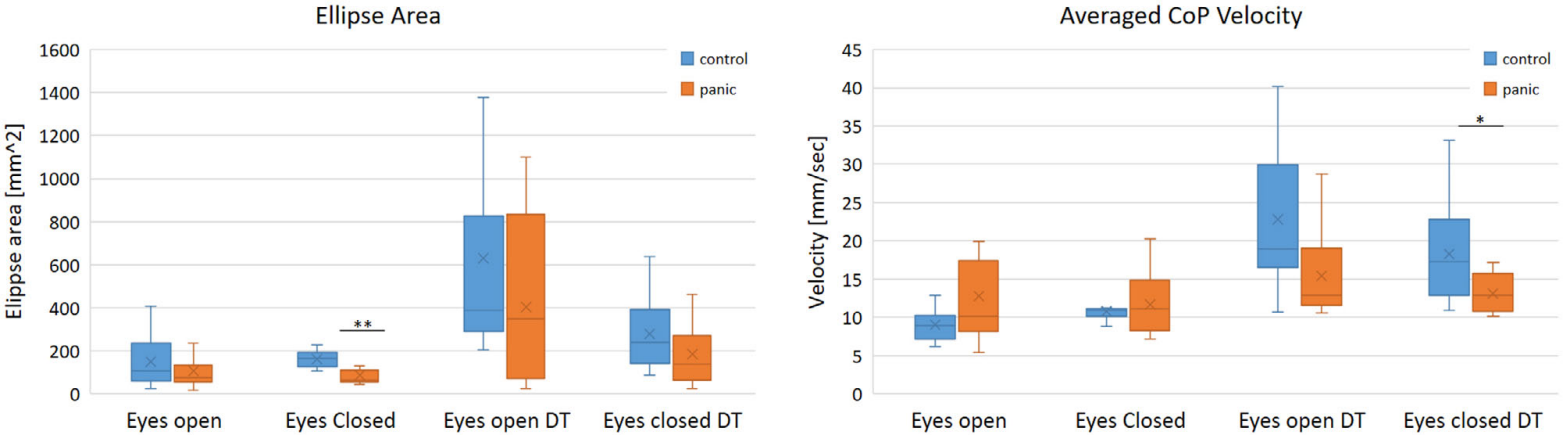
Box plots of Dc_o_p ellipse area (left) and of velocity (right) in four sway conditions depicted for both groups (see color key). Within each box, horizontal lines denote median values and “X” markers denote mean values; boxes extend from the 25th to the 75th percentile of each group's distribution of values; vertical extending lines denote adjacent values (i.e., the most extreme values within 1.5 interquartile range of the 25th and 75th percentile of each group). *p < .05; **p < .01

Interestingly, both Dc_o_p and $\bar{V_{\mathrm{CoP}}}$ parameters of sway were decreased in transition from EO to EC under both DT and NoDT conditions in the PD group, whereas in the control group, similar behavior was observed only under the DT condition (Table 2, Figure 3).

Adding DT increased Dc_o_p and $\bar{V_{\mathrm{CoP}}}$ sway parameters in both EO and EC conditions. This effect was generally smaller in the PD compared to the control group, but a significant group effect was found only for the $\bar{V_{\mathrm{CoP}}}$ in EC condition and for DTC of the$\;\bar{V_{\mathrm{CoP}}}$ in the EO condition. One to two outliers were removed from the analysis of static balance performances (see details in the legend of Table 2).

Post hoc analyses to detect potential task prioritization effect as the source of the differences in DT conditions based on C‐DTC values (see Section 3) revealed no between‐group effects (*p* ≥ .53; see details in Table 3).

**TABLE 3:** Performance on cognitive task and cognitive dual task cost (C‐DTC)

	Parameter	Healthy [mean±SD]	PD [mean±SD]	P value*
**Counting rate (CR)** [counts/min]	Reference	17.5 ± 3.6	11.9 ± 3.6	**0.0139**
	Sway (EO)	19.9 ± 4.5	12.9 **± **4.2	**0.0085**
	Perturbations	16.3 **± **3.8	9.7 **± **4.8	**0.0198**
**Percent Change C‐DTC** [%]	BS vs. Sway	−13.5 ± 9.8	−17.4 ± 21.4	N.S (0.6672)
	BS vs. Pert	9.5 ± 7.6	9.9 ± 31.6	N.S (0.9601)
	Sway vs. Pert	17.1 ± 12.7	24.0 ± 21.8	N.S (0.5287)

* Non‐parametric Mann‐Whitney *U*‐test between panic disorder (PD) and healthy participants.

CR‐ counting rate; EO – eyes closed; C‐DTC – cognitive dual task cost.

### 3.3 Dynamic measurement

Small percentage of perturbations was discarded (2.0%) due to participants’ failure to regain stability before the consecutive perturbation took place and due to other technical problems.

In about a third of the perturbations, participants from both groups reacted with a compensatory step (e.g., single step, double step, and multiple steps). However, taking multiple steps to recover from a perturbation was significantly more frequent in the PD group than in healthy controls (Table 2), reflecting a less stable and ineffective recovery strategy (Brauer et al., 2002).

It was found that participants with PD deviated significantly faster than healthy controls when an in‐place response was used, and recovered faster from the perturbations when a step response was used. No significant DT effect was found regarding the ability to regain stability following perturbations in either group. Likewise, Dc_o_p and $\bar{V_{\mathrm{CoP}}}$ were not significantly different between groups in the standing periods prior to perturbations (Table 2).

### 3.4 Effect of agoraphobia

In our pilot study, seven of 11 patients suffered from agoraphobia. No differences were found in any of the balance parameters between the agoraphobia and nonagoraphobia subgroups (data not shown).

## 4 DISCUSSION

### 4.1 Summary of the results

Evaluating balance control in PwPD using dynamic physical perturbation is feasible and could provide objective balance measures. In our assessment we used balance clinical rating scales to identify potential differences between the PD and control groups (e.g., DHI and ABC scale) (Balaban & Thayer, 2001; Caldirola et al., 2011; Jacob & Furman, 2001; Vaillancourt et al., 2012; Yardley et al., 1995, 2001). Participants with PD demonstrated lower scores on all tests (Table 1) in agreement with previous results (Asmundson et al., 1998; Caldirola et al., 2011; Jacob et al., 1997; Perna et al., 2001; Staab, 2006).

Further, and in agreement with earlier observations (Levitan et al., 2012; Lopes et al., 2009), the present study observes that PwPD adopted a rather stiff standing that was expressed in smaller sway area, higher $\bar{V_{\mathrm{CoP}}}$ and faster reactions to perturbations compared to healthy controls (c.f. Table 2 and Figure 3). This also corroborates the balance reactivity of healthy participants showing high anxiety levels (Adkin & Carpenter, 2018; Cleworth & Carpenter, 2016; Okada et al., 2001). As expected, PwPD exhibited higher anxiety levels (Table 1).

### 4.2 Balance control mechanisms adopted by PwPD—Results from computerized assessments

#### 4.2.1 Static sway tests with open and closed eyes

Overall, PwPD exhibit immobility and postural “rigidity.” This is implied by their tendency to have smaller sway areas in all four conditions, compared to controls. Furthermore, they reduce their sway area in the transition from open to closed eyes state, while the controls responded contrariwise (i.e., increase their sway area), as expected (Macedo et al., 2015) (see Table 2). In addition, we also observed a tendency of PwPD to have higher $\bar{V_{\mathrm{CoP}}}$ compared to controls, similar to what has been shown in the literature (Caldirola et al., 2011; Perna et al., 2001). Similar behavior, which was previously shown in the study of Lopes et al. (2009), suggested that PwPD adopt a strategy of postural "rigidity” and attributed this “choice” to the increased background levels of anxiety. Thus, when PwPD close their eyes, “freezing/halting” behavior predominates the natural tendency to increase sway (Lopes et al., 2009). PwPD is not the only population with balance impairments to adopt this behavior. Similar results were seen among children with autism (Gepner et al., 1995), who exhibited smaller sway areas in the EC condition. These results are also in agreement with the conclusion of Adkin and Carpenter's review (2018) that points to the same behavior by healthy participants with high level of anxiety.

#### 4.2.2 Static sway tests in the presence of cognitive load (DT)

Our finding that PwPD adopt postural "rigidity” is further supported by the results from the DT trials. During DT, both groups were challenged by the need to divert attention to the subtraction task. However, during EO conditions DTC was significantly smaller among PwPD as compared to healthy controls with regards to the $\bar{V_{\mathrm{CoP}}}$ parameter (Table 2). We speculate that due to the "rigidity" strategy, governing the balance control function among PwPD DT effects is attenuated. High anxiety level and "rigidity" strategy prevent PwPD from modifying their balance behavior according to the challenging situation, as done by healthy controls (i.e., controls have higher DTC). It was hypothesized that an increase in sway and $\bar{V_{\mathrm{CoP}}}$ during DT in healthy participants points to the fact that both cognitive and balance controls are funneled through common neuronal resources (Tombu & Jolicoeur, 2003; Yogev‐Seligmann et al., 2008). It appears that the control system weighs out between the two congruent tasks and thus compromises one (or both) to optimize its response. The fact that no between‐group task prioritization effect was found further supports our speculations.

#### 4.2.3 Responses to perturbations—Dynamic assessment

Similar to the static measurement, the computerized dynamic balance results showed that PwPD had higher $\bar{V_{\mathrm{CoP}}}$ than controls during the in‐place response. When stepping response was used, PwPD recovered faster from perturbations, as compared to controls. Both groups demonstrated similar prevalence of stepping responses; however, it appears that when doing so, PwPD more often generate a multiple steps response, perhaps indicating more hesitative behavior and lack of confidence that “one” step is sufficient to recover their balance.

Unlike static sway, dynamic conditions such as those imposed during the perturbation trials challenge the body balance and increase anxiety and fear (Horslen et al., 2013). Therefore, possibly due to their background anxiety, PwPD recover even faster than controls, mainly by using higher $\bar{V_{\mathrm{CoP}}}\;$and multiple stepping responses (Table 2). This strategy may reflect a nonsecure compensatory behavior of PwPD (Miller et al., 1989). Studies performed on healthy participants exposed to high anxiety terms show faster response of the body and higher $V_{\mathrm{CoP}}$ as anxiety levels increased (Carpenter et al., 2004; Cleworth et al., 2018; Okada et al., 2001).

It is worth noting that both groups performed similarly during the standing periods prior to the introduction of perturbations and demonstrated larger Dc_o_p compared to static sway (Table 2), corroborating previous results that show larger Dc_o_p when anticipating perturbations (Bax et al., 2020; Johnson et al., 2019a, 2019b). However, we suggest that higher $\bar{V_{\mathrm{CoP}}}$ in response to perturbations seen among PwPD can still be interpreted as reflecting "rigidity" strategy (Caldirola et al., 2011; Redfern et al., 2007; Stambolieva & Angov, 2010). This point warrants further investigation since the prior perturbations standing periods used in this study were rather short (5 s).

Interestingly, the DT condition showed no group effect in the measurements of the dynamic balance performance. It was previously suggested by Redfern et al., who has observed similar results, that since recovery from perturbation is a relatively strong automated postural adjustment, the response is not evidently affected by simultaneous, relatively simple, and cognitive tasks (Redfern et al., 2002).

### 4.3 Evaluating the computerized balance tests’ results in light of the functional tests

More pronounced group effect was seen in the comparisons related to the functional tests, as compared to the computerized tests. This outcome points to the different perspective of postural control, objective computerized technology and clinical balance rating scales, providing additional subtleties when attempting to understand balance control mechanisms (Mancini & Horak, 2010).

Some of these differences can be attributed to psychological factors. For example, PwPD tend to subjectively complain more about balance impairments than normal controls, as shown by Teggi et al., who compared two groups' chronic dizziness, with and without PD (Teggi et al., 2007). In the present study, the PD group complained more about dizziness symptoms and had higher DHI scores, maybe due to the underestimation of self‐abilities.

In this regard, of relevance is a recent study that has found that experimentally‐induced anxiety leads to distorted perceptions of subjective instability (Ellmers et al., 2021), which may lead us back to adopting over all "rigid" postural behavior.

Taken together, the results of this study suggest that PwPD have different balance characteristics than healthy controls. In addition to the psychological effects that draw individuals suffering from anxiety towards choosing a more “rigid” strategy for maintaining balance, it has been suggested that physiological functions are also involved in these differences, such as vestibular function (Feldman et al., 2019).

### 4.4 Conclusions, study limitations, and future work

The main limitation of this pilot study is that it was underpowered (see Section 2). Furthermore, there was a small number of data points for both groups due to missing data. For instance, DT effects, which are more variable, may have been harder to detect. Another limitation was that nine PwPD took psychotropic medications, which have an adverse effect of dizziness. On the other hand, these medications can also reduce dizziness. Four patients took medications that are known to have relatively high prevalence of dizziness (e.g., oxazepam 10 mg or clomipramine), and five patients took medications with low prevalence of dizziness (e.g., mirtazapine or escitalopram). These psychiatric medications have a long‐term effect; thus, assessing PwPD with the bias of medication effects is unavoidable.

In addition, unlike previous studies, our sample of PwPD included patients from our psychiatric clinic without inclusion criteria of dizziness or balance disturbances. In fact, we did not obtain clinical information about their vestibular function from otolaryngology clinics, while earlier studies have contrasted their results with this factor (Clark et al., 1994; Jacob & Furman, 2001; Staab & Ruckenstein, 2003).

With regards to our findings, it is also worth noting that in the present protocol we did not assess within‐task anxiety levels (i.e., state anxiety). This limits our ability to draw conclusions about the adoption of "rigidity" strategy among PwPD. It has been previously described that persons traits influence the postural control strategies adopted under height‐induced postural threats (Zaback et al., 2015). Complementary work is warranted to assess the state of anxiety simultaneously with the postural tasks.

## CONFLICT OF INTEREST

The authors declare no conflict of interest.

### PEER REVIEW

The peer review history for this article is available at https://publons.com/publon/10.1002/brb3.2411

## Data Availability

Data will be available by the corresponding author upon reasonable request.
